# DNA polymerase I and DnaE mediate RNA-templated DNA synthesis over embedded ribonucleotides

**DOI:** 10.1093/nar/gkag971

**Published:** 2026-10-08

**Authors:** Frances C Lowder, Abigail H Kendal, Lyle A Simmons

**Affiliations:** Department of Molecular, Cellular, and Developmental Biology, University of Michigan, Ann Arbor, MI 48109, United States; Department of Molecular, Cellular, and Developmental Biology, University of Michigan, Ann Arbor, MI 48109, United States; Department of Molecular, Cellular, and Developmental Biology, University of Michigan, Ann Arbor, MI 48109, United States

## Abstract

It is established that during normal growth and under stress, ribonucleotides become nested in genomic DNA. Given the high frequency with which RNA is found in DNA, it is unknown which bacterial DNA polymerases are responsible for catalyzing RNA-dependent DNA synthesis. In this work, we establish bacterial DNA polymerase I (Pol I), a robust reverse transcriptase (RT), as the primary bypass enzyme. We tested several bacterial Pol I enzymes and found that all possess RT activity, with *Bacillus subtilis* Pol I showing robust RNA-dependent DNA synthesis activity similar to the Moloney murine leukemia virus RT. In contrast, template RNA slowed or blocked DNA synthesis by *B. subtilis* replicative C-family DNA polymerases PolC and DnaE. We tested several substrates with increasing patches of consecutive ribonucleotides in DNA and found that a single ribonucleotide slows synthesis by PolC, while DnaE can traverse a patch of five consecutive ribonucleotides. Therefore, DnaE activity would allow for ribonucleotide bypass of short patches, while Pol I could bypass longer stretches up to 40 nt in length. We conclude that Pol I and DnaE are responsible for RNA-templated DNA replication, establishing the DNA polymerase hierarchy for navigating RNA tracts that become integrated into genomic DNA.

## Graphical Abstract

**Graphical Abstract:**
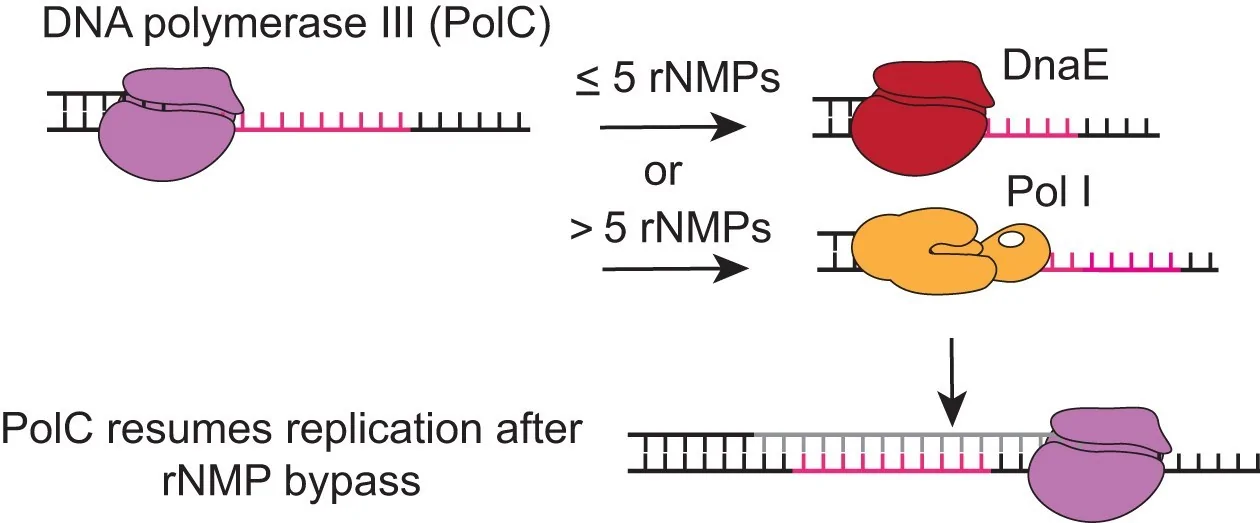

## Introduction

RNA–DNA hybrids represent one of the most prevalent and pervasive endogenous nucleic acids that lead to genome instability in all organisms (summarized in Fig. 1A, reviewed in [1]). Although RNA–DNA hybrids are well documented for their detrimental effects on genome integrity, hybrid formation is essential for all cells due to the role of hybrids in priming DNA synthesis [2, 3] and in the production of messenger RNA during transcription [4, 5]. RNA–DNA hybrids also contribute to DNA repair during suboptimal conditions [6, 7] and regulate gene expression [8]. Failure to resolve hybrids is a driving force for genome instability in organisms ranging from bacteria to humans and underlies the development of several human cancers and neurological disorders [9, 10]. The nucleolytic activity of the 2′-OH of the ribose sugars can nick the phosphodiester backbone of nearby DNA, leading to the formation of DNA strand breaks [11]. Persistent RNA–DNA hybrids resulting from RNA polymerase impose barriers to DNA replication, causing fork stalling [12–15], induction of the DNA damage response [16], hyper-recombination and genome rearrangements [17, 18]. All of these outcomes threaten cell survival and fitness, and have the potential to lead to the development of human diseases [19, 20] and antibiotic resistance [21, 22].

**Figure 1.**
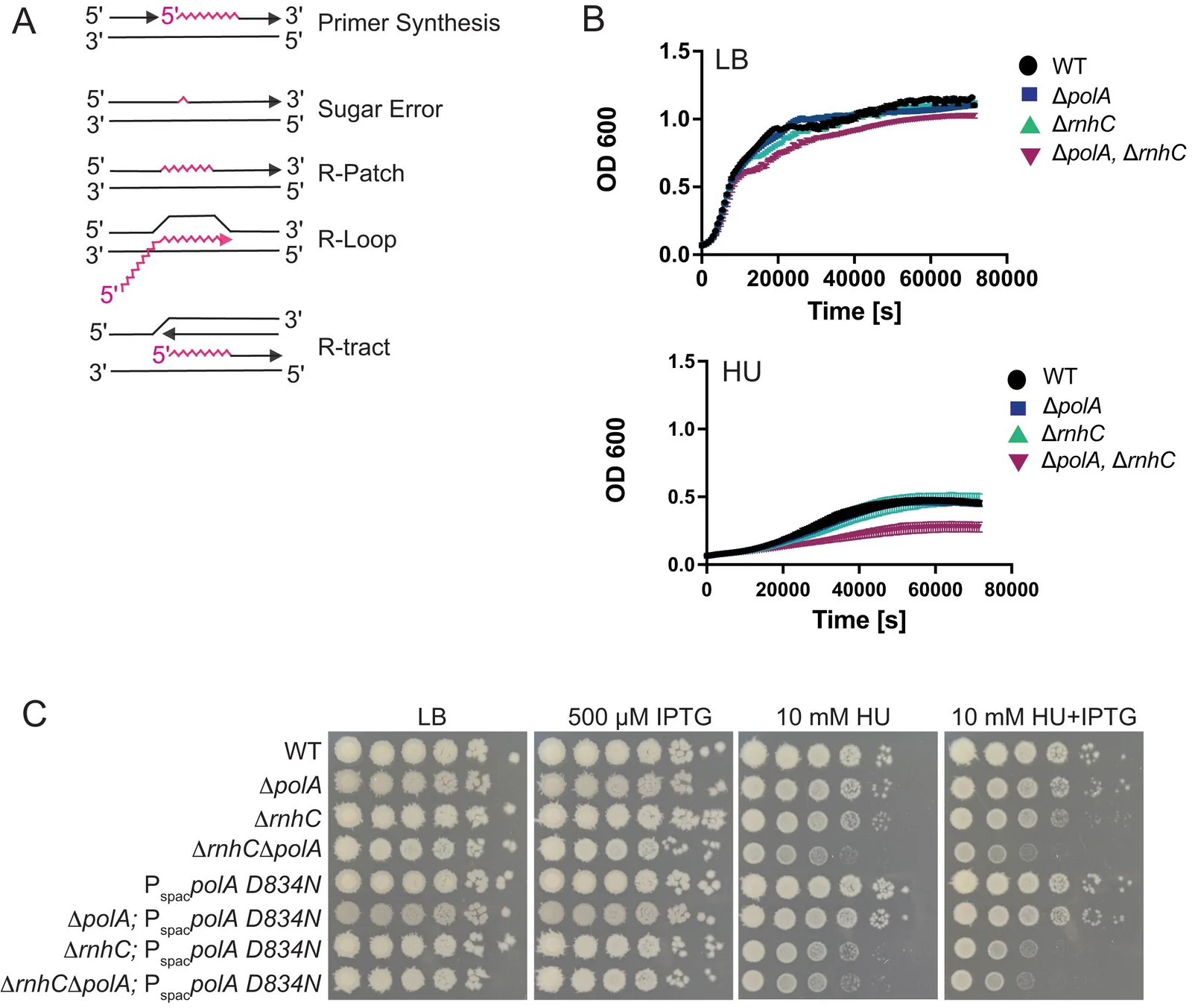
The absence of *Bs* Pol I sensitizes cells to ribonucleotide incorporation. (**A**) Depictions of the major types of RNA–DNA hybrids. The black lines correspond to DNA, the magenta squiggly lines correspond to RNA. (**B**) Growth curves of the indicated strains in the presence or absence of hydroxyurea (HU). HU in liquid culture was used at 25 mM. Error bars represent standard error of the mean. (**C**) Spot titers of *Bacillus subtilis* deletion strains treated with HU.

It is well established that replicative DNA polymerases from bacteria to eukaryotes misincorporate ribonucleotides (rNMPs) in place of their cognate deoxyribonucleotides, forming a “sugar error” [23–27]. Sugar errors are a major source of RNA–DNA hybrids that become embedded in the genome, as rNMP errors occur on the order of a couple thousand per round of replication for a bacterium [25] to over a million per round of replication for mammals [23, 27]. Should ribonucleotide errors persist in the template strand, they can slow or possibly arrest progression of replicative bacterial DNA polymerase III [25, 28, 29] and eukaryotic replicative DNA polymerase δ and polymerase ε, though these latter two can efficiently bypass ∼1 rNMP [30–32]. In addition to single ribonucleotide errors, stretches of four or more rNMPs can be incorporated into genomic DNA. These include R-patches, which arise from errors by translesion DNA polymerases [7, 33], and R-tracts, which result from the integration of R-loops formed during transcription [34]. Such extended stretches of RNA–DNA hybrids pose significant problems for DNA replication [25, 27, 30]. When left unrepaired, cells would need to use a reverse transcriptase (RT) to catalyze RNA-templated DNA synthesis across the embedded RNA, or the replication fork would arrest [35]. Failure to bypass or repair the embedded ribonucleotides would cause fork stalling and activation of the DNA damage response, the mutagenic consequences of which threaten genome integrity and can lead to antibiotic resistance in bacteria [21, 22].

A-family DNA polymerases are well represented among bacteria, viruses, bacteriophages, and eukaryotes. The archetypal A-family DNA polymerase is *Escherichia coli* DNA polymerase I (Pol I) [36]. In addition to DNA synthesis activity, *E. coli* Pol I has 5′–3′ exonuclease activity and 3′–5′ proofreading exonuclease activity, and as such it performs high-fidelity DNA synthesis [37–39]. Furthermore, it has been shown that full-length *E. coli* Pol I, the Klenow fragment, and Family A enzymes (Pol I) from thermophiles can synthesize DNA from an RNA template with Mn^2+^ often used to enhance activity [40–43]. The eukaryotic DNA polymerase, Pol θ, is able to traverse rNMPs embedded in template DNA [44, 45]. Pol θ is another A-family DNA polymerase that has an inactive proofreading domain [44, 45], is highly error-prone, and has also been shown to catalyze RNA-templated DNA synthesis [46]. However, Pol θ RT activity is inefficient relative to the Pol I Klenow fragment [47]. Therefore, although Pol I from *E. coli* can synthesize DNA from an RNA template [40, 41], most studies showing RT activity from a Pol I or similar A-family polymerase have used proofreading-defective enzymes from thermophilic species [40, 41]. Further, although RT activity has been described for several Pol Is [40–43], a phenotype linking RT activity to synthesis over an RNA template has not been shown, causing the *in vivo* relevance for Pol I RT activity to remain unclear.

There are numerous studies of *E. coli* Pol I, several of which are foundational to our understanding of DNA replication [36]. Because of this, *E. coli* Pol I has long been the model for bacterial Pol I enzymes [48]. In contrast to *E. coli*, a Gram-negative bacterium, many Gram-positive bacteria have evolved a Pol I enzyme that lacks 3′–5′ proofreading exonuclease activity, due to mutations that prevent coordination of the Mg^2+^ required for catalysis [49, 50]. This vestigial proofreading domain of Gram-positive Pol I is reminiscent of the inactive proofreading domain of eukaryotic Pol θ, which allows for Pol θ to become error-prone and may contribute to RNA-templated DNA synthesis [45, 51]. Consequently, it remains unexplored as to whether Gram-positive Pol I enzymes have innate RT activity and could participate in RNA-templated DNA synthesis over unrepaired ribonucleotides that accumulate in genomic DNA. Further, it is unknown if replicative C-family DNA polymerases from Gram-positive bacteria can tolerate ribonucleotides in the template strand and, if so, how many. Therefore, the division of labor used by bacterial DNA polymerases to bypass embedded ribonucleotides remains unknown.

Here, we investigated the impact of ribonucleotide-containing templates on the DNA polymerase activity of both A- and C-family DNA polymerases from the Gram-positive bacterium *B. subtilis*. We found that an RNA template blocks or slows synthesis by replicative C-family DNA polymerases PolC and DnaE. In contrast, we found that *B. subtilis* Pol I (*Bs* Pol I) is capable of robust RT activity, demonstrating the potential for RNA-templated DNA synthesis activity. We show that Pol I traverses DNA templates containing consecutive ribonucleotides, demonstrating that Pol I is able to replace a replicative DNA polymerase when a ribonucleotide patch is encountered. We expanded our findings and tested several other bacterial Pol I enzymes and found that many have innate RT activity, albeit with varying efficiencies. We demonstrate that ribonucleotide incorporation in DNA represents a source of genome instability that is exacerbated in the absence of Pol I DNA polymerase activity, underscoring the importance of RT activity *in vivo*. Further, we show that the C-family enzyme DnaE can bypass up to five consecutive rNMPs, demonstrating two pathways for replication over ribonucleotide lesions. Together, this study demonstrates the critical contribution of bacterial Pol I and DnaE and establishes a hierarchy of DNA polymerases used for RNA-templated DNA synthesis activity to bypass ribonucleotides embedded in genomic DNA.

## Materials and methods

### Reagents

M-MuLV (M0253S) and *E. coli* Pol I (M0209S) were purchased from New England Biolabs.

### *Bacillus subtilis* expression of *polAD834N*

The *polAD834N* gene was constructed using site-directed mutagenesis of plasmid pFCL23, a pDR110 derivative, which contains the *polA* gene [54]. Plasmid pDR110 allows for double-crossover integration at *amyE* with the gene of interest under isopropyl β-D-1-thiogalactopyranoside (IPTG)-inducible *P*_spank_ control. Mutagenesis was conducted using overlapping primers prFCL182 and prFCL183 (Supplementary Table S2) to change the D codon (GAT) to N (AAC). Polymerase chain reaction (PCR) amplification of pFCL23 generated plasmid pFCL93. Plasmid pFCL93 was then used to transform *B. subtilis* WT strain PY79 using standard procedures [55], generating strain FCL263. Chromosomal DNA from this strain was isolated and used to transform additional strains as shown in Supplementary Table S1.

### Spot titers

*Bacillus subtilis* strains (Supplementary Table S1) were streaked onto LB plates and incubated overnight at 37°C. For each strain, 2 ml of LB was inoculated with a single colony and then grown at 37°C in a rolling rack until A_600nm_ was between 0.5 and 0.7. Cultures were pelleted by centrifugation (4000 × *g*/5 min/25°C), and the supernatant was removed and replaced with 0.85% w/v NaCl (saline). Samples were normalized to A_600 nm_ = 0.5, then serially diluted to 10^−5^ in saline. Four microliters of each dilution was plated on LB agar with or without 10 mM HU, then grown for 12 h at 37°C. Spot titers were performed in biological triplicate, and plates were prepared on the day of the experiment to minimize HU degradation [52]. Image brightness and contrast were adjusted in FIJI [53].

### Growth curves

The appropriate *B. subtilis* strains (Supplementary Table S1) were streaked onto LB plates and incubated overnight at 37°C. For each strain, a single colony was used to inoculate 2 ml of LB, which was grown at 37°C in a rolling rack until A_600 nm_ was between 0.5 and 0.7. The culture was diluted to A_600 nm_ using LB or using LB with 25 mM HU in a 96-well plate with a clear bottom panel and lid lifter. A_600 nm_ values were collected every 600 s for 20 h for each well using a Tecan Spark plate reader set to 37°C with continuous shaking. Four measurements of each well were taken per cycle. Three biological replicates were performed for each condition, where each consisted of two technical replicates.

### Expression plasmid cloning

All primers used are listed in Supplementary Table S2, with all plasmids described in Supplementary Table S3.

The protein sequences for LmPolA (UniProt ID: A0A6Y7VQD1) and MsPolA (UniProt ID: I7G3P9) were submitted to Twist Bioscience for gene synthesis and insertion into the pTwist vector harboring Kan resistance. Inserted genes were codon-optimized for expression in *E. coli* and, to simplify downstream cloning, 25 bp of the pE-SUMO vector was added upstream and downstream of the gene.

Ec*polA* (UniProt ID: P00582) and Sa*polA* (UniProt ID: A0A0H2XGR4) were amplified using genomic DNA from *E. coli* BL21 (DE3) or *Staphylococcus aureus* USA300 (genomic DNA from Dr Taylor Nye) as appropriate, while *LmpolA* and *MspolA* were amplified from their respective pTwist plasmids. Empty, linear pE-SUMO expression vector was amplified using oJR46 and 47.

After gel extraction, each insert was combined with vector using the NEB HiFi protocol (NEB E2621). Assembly reactions were used to transform chemically competent DH5α cells, recovered at 37°C for 1 h, and grown overnight at 37°C on LB agar supplemented with 25 μg/ml kanamycin. Transformants were screened using colony PCR with prFCL124 and 125, then the plasmid sequence was confirmed with full-plasmid sequencing.

### Protein expression

Chemically competent *E. coli* BL21 (DE3) were transformed with plasmid (Supplementary Table S4), plated on LB + Kan (25 μg/ml kanamycin, unless stated otherwise), and grown overnight at 37°C. Starter culture was prepared by inoculating LB + Kan with transformant cells, which was then grown overnight at 37°C while shaking. For expression, each liter of LB + Kan was inoculated with starter culture, then grown to A_600 nm_ ∼ 0.7. Cultures were induced by adding IPTG to 0.5 mM, then grown for 3 h at 37°C. Induced cells were harvested by centrifugation at 4000 × *g*/4°C/25 min, after which the supernatant was discarded and pellets were stored at −80°C.

### Protein purification

All purification steps were performed at 4°C. Cell pellets were thawed on ice, then resuspended in lysis buffer (20 mM Tris, pH 8.0, 400 mM NaCl, 5% v/v glycerol), supplemented with protease inhibitor (Pierce: A32965) and 1 mM phenylmethanesulfonyl fluoride. Cells were lysed via sonication, and then the lysate was clarified by centrifugation at 30000 × *g* for 30 min. Clarified lysate was applied to a gravity column containing Ni^2+^-NTA-agarose that had been pre-equilibrated in lysis buffer and allowed to pass through. The column was washed with 5 CV lysis buffer, then with 5 CV of wash buffer (20 mM Tris, pH 8.0, 2 M NaCl, 15 mM imidazole, 5% v/v glycerol). Protein that remained bound to the column was eluted with 3 CV elution buffer (20 mM Tris, pH 8.0, 400 mM NaCl, 300 mM imidazole, 5% v/v glycerol). The wash and elution fractions were evaluated using sodium dodecyl sulfate–polyacrylamide gel electrophoresis (SDS–PAGE), then the protein-containing fractions were pooled and treated with Ulp1 protease for 2 h at room temperature to remove the 6×-His-SUMO tag. The protein was then dialyzed overnight against dialysis buffer (20 mM Tris, pH 8.0, 300 mM NaCl, 5% v/v glycerol). Dialyzed protein was then passed over Ni^2+^-NTA-agarose that had been equilibrated with dialysis buffer. The column was washed with 3 CV dialysis buffer, and all flowthrough was collected and diluted to 50 mM NaCl with Q buffer A [20 mM Tris, pH 8.0, 5% v/v glycerol, 1 mM dithiothreitol (DTT)]. This protein was filtered with a 0.22 µm syringe filter and applied to a HiTrap Q FF anion-exchange column (Cytivia, 17515601) and fractionated with an AKTA FPLC. Protein was eluted using an increasing gradient of Q buffer B (20 mM Tris, pH 8.0, 500 mM NaCl, 5% v/v glycerol, 1 mM DTT). Peak fractions were evaluated using SDS–PAGE, then pooled and concentrated using a centrifugal filter. Glycerol was added to 25%, and then proteins were aliquoted, flash-frozen, and stored at −80°C. The gel demonstrating the proteins used in this paper was made by loading 2 µg of each protein onto a Mini-PROTEAN TGX 4%–20% gradient gel (Bio-Rad, 4561096) and then staining with Coomassie blue.

### Extension assays

Extension assays were carried out as described previously [54, 56]. In brief, annealed oligonucleotide substrates were generated by combining 1 µM of the primer and 2 µM of the template in 1× dilution buffer (20 mM Tris, pH 8.0, 25 mM NaCl), then heating them at 98°C for 1 min. Substrates were allowed to cool to room temperature. All oligonucleotides used were purchased from IDT, and sequences are included in Supplementary Table S2. Substrates were made using the following oligonucleotide combinations. The DNA templates used in Figs 2 and 3 use oFCL7 and 8, with oFCL27 as the ladder. The short RNA templates used in Figs 2 and 3 and Supplementary Fig. S2 use oFCL16 and oJR227, with oFCL20 as the ladder. The long RNA templates used in Figs 2 and 3 use oFCL18 and oJR336. Embedded ribonucleotide substrates (Fig. 4 and Supplementary Figs S3 and S4) consist of oFCL21 annealed to oFCL22, 23, 24, 25, or 26.

For each reaction, a new aliquot of protein was diluted to 400 nM in 1× Metals Buffer (20 mM Tris, pH 8.0, 50 nM NaCl, 1 mM MgCl_2_). Protein was added, at a final concentration of 100 nM, to a reaction mix containing 100 nM substrate, 50 µM deoxynucleotide triphosphates (dNTPs), and 1× Extension Buffer (40 mM Tris-acetate, pH 7.8, 12 mM magnesium acetate, 300 mM potassium glutamate, 3 μM ZnSO_4_, 2% (w/v) polyethylene glycol, 0.02% pluronic F68) or 1× RT Buffer (50 mM Tris, pH 8.0, 75 mM KCl, 3 mM MgCl_2_). The RT Buffer was based on the 1× M-MuLV RT Reaction Buffer (New England Biolabs, B0253S). M-MuLV (M0253S) and *E. coli* Pol I (M0209S) controls were purchased from New England Biolabs. Commercial protein concentrations were estimated using NanoDrop spectrophotometry and were used at the same concentrations as the other proteins, which translates to 2.4 units of M-MuLV and 0.15 units of *Ec*Pol I per reaction. Assays were performed at 37°C for 60 min, except for those using the embedded ribonucleotide substrates, which were incubated at 37°C for 20 min (Fig. 4) or for the time indicated (Supplementary Fig. S4). Reactions were terminated by adding an equivalent volume of Stop Buffer (95% formamide, 20 nM ethylenediaminetetraacetic acid, bromophenol blue) to the reaction, boiling them for 10 min at 98°C, and then snap-cooling in ice. Reaction products were resolved by Urea-PAGE using gels containing 20% acrylamide and 8 M urea. To minimize the persistence of nucleic acid complexes, the 1× TBE (Tris-Borate-EDTA) running buffer was pre-warmed to ~45°C and the electrophoresis box was kept on a 45°C hot plate while voltage was applied. Gels were visualized using the 700 and 800 nm channels of a LiCor Oddesey CLx, and channel color was altered from red/green to magenta/green using FIJI [53]. Original colors are included in Supplementary Table S2.

## Results

### The absence of *Bs* Pol I sensitizes cells to ribonucleotide incorporation

There is substantial evidence that ribonucleotides are embedded in genomic DNA [7, 31, 57–61], and although some Pol Is can use an RNA template [40–43], a phenotype establishing the relevance of RT activity *in vivo* has not been defined. Therefore, we asked whether loss of Pol I sensitized *B. subtilis* cells to increased ribonucleotide concentrations *in vivo*. To explore this question, we challenged cells with HU, a compound that inhibits ribonucleotide reductase and increases the occurrence of RNA–DNA hybrids, particularly sugar errors and R-patches (Fig. 1A) [52]. We also inactivated the *rnhC* gene to increase the occurrence of R-loops in *B. subtilis*, which could cause conversion of R-loops to R-tracks [62].

As shown in Fig. 1B, Δ*polA* and Δ*rnhC* cells grow similar to WT in the presence or absence of HU in liquid medium. When cells with the double mutant (Δ*polA*Δ*rnhC*) were incubated in the presence of HU, they showed reduced growth (Fig. 1B). We performed a similar test on solid medium. As with the growth curves, WT, Δ*polA*, and Δ*rnhC* cells grew similarly on LB or in the presence of HU, where Δ*rnhC* shows the least amount of growth. With a Δ*polA*Δ*rnhC* strain, we observed growth interference in the presence of HU (Fig. 1C). To test whether the increase in sensitivity of the double mutant was due to a lack of DNA polymerase activity from Pol I, we tested a catalytic mutant *D834N* ectopically expressed from the *amyE* locus in the chromosome. We found that ectopic expression of *polA (D834N)* grew the same as WT whether the native *polA* gene was intact or deleted, which is due to the presence of a secondary FEN that can substitute for the absence of the 5′–3′ exonuclease activity of Pol I [54]. When *polA (D834N)* was combined with Δ*rnhC* or Δ*rnhC*Δ*polA*, we observed the same phenotype as the Δ*rnhC*Δ*polA* double mutant strain. These results show that the hypersensitivity to HU of Δ*polA* in an Δ*rnhC* background is caused by a lack of DNA polymerase activity conferred by the Δ*polA* allele. This result underscores the importance of Pol I DNA polymerase activity in responding to RNA–DNA hybrids in *B. subtilis* and provides a phenotype for Pol I RT activity. With these results, we suggest that the sensitivity of the Δ*polA* and *polA (D834N)* strains in an Δ*rnhC* background is caused by a failure of replication over RNA nested in the template strand.

### *Bacillus subtilis* Pol I can use RNA as a synthesis template

While Pol I is strongly associated with DNA repair, some Pol Is have also been shown to have RT activity [40–43]. As such, we were interested in testing the *B. subtilis* DNA polymerases for RNA-templated DNA synthesis. Because *polC* and *dnaE* are essential genes [56, 63], we used a biochemical approach to determine if DNA polymerases could synthesize DNA using a template comprised entirely of ribonucleotides. Using purified Pol I, PolC, and DnaE (Supplementary Fig. S1), we assayed primer extension using a DNA or RNA template. As a control, we also assayed the known RT from Moloney Murine Leukemia Virus (M-MuLV) [64]. Figure 2A demonstrates that all three *B. subtilis* polymerases were catalytically active, with robust activity shown using a DNA template in a buffer optimized for *B. subtilis* replication [56] (Ext. Buffer). All three polymerases were also active in a simpler buffer used to promote RT activity (RT Buffer); however, there were minor differences in the final products generated (Fig. 2B). Note that the RNA template migration is slower than the DNA product, as slower RNA migration is attributed to the properties of RNA relative to DNA. When the template strand was composed entirely of RNA, Pol I was the only DNA polymerase able to fully traverse RNA and generate the same product as the RT control in either buffer (magenta band) (Fig. 2C and D). Conversely, PolC produced consistent intermediate products using the RNA template, while synthesis by DnaE was completely blocked (Fig. 2C and D). If the RNA template was twice as long, Pol I still produced similar products to the control RT, while PolC and DnaE could only perform minimal primer extension (Fig. 2E and F). We conclude that wild-type Pol I from *B. subtilis* is a robust RT, and that replicative C-family polymerases are poor at engaging RNA-templated DNA synthesis in comparison to Pol I. Further, we note that Pol I can perform RNA-templated DNA synthesis under different assay conditions (Ext. or RT Buffer), suggesting that RT activity is not dependent on a specific *in vitro* experimental design, supporting the conclusion that RT activity is an authentic property of *B. subtilis* Pol I.

**Figure 2.**
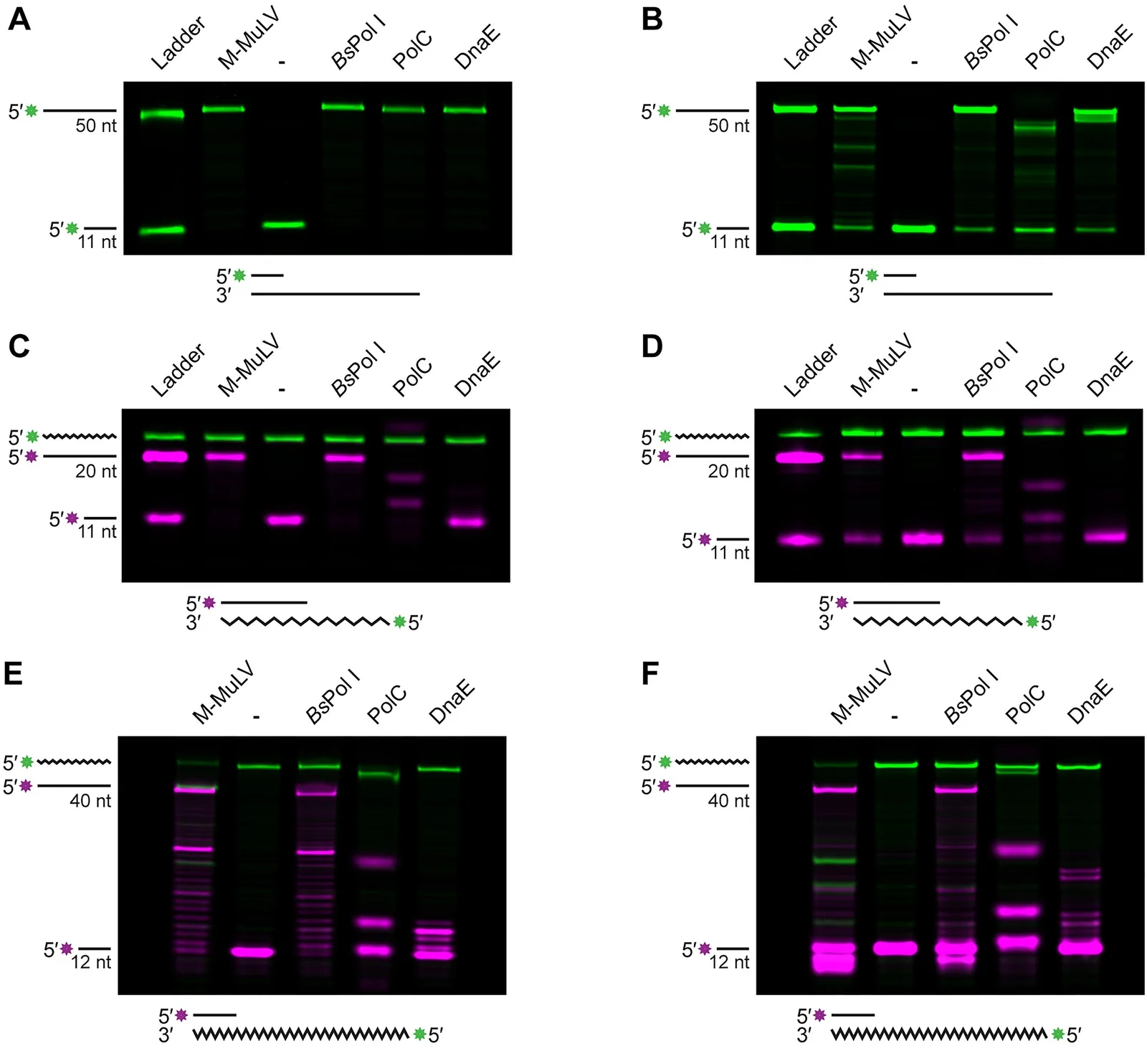
*Bs* Pol I can use RNA as a synthesis template. (**A, B**) Primer extension using a 50-nt DNA template, (**C, D**) a 20-nt RNA template, and (**E, F**) a 40-nt RNA template. A, C, and E used a buffer optimized for DNA polymerase extension (Materials and methods). B, D, and F are the same except reactions used a buffer optimized for RT activity (Materials and methods).

### RNA-templated DNA repair is conserved in bacterial Pol I enzymes

Based on our biochemical results, we asked if the RT activity we observed for *Bs*PoI I was conserved among other bacterial Pol I enzymes. We compiled a list of other instances where Pol I enzymes had been shown to have RT activity (Supplementary Table S4). Given the variety of experimental conditions in these works including high levels of Mn^2+^ [42, 43], no clear comparison can be made from this data; however, several trends are noted. Of the 22 bacterial Pol Is shown to have RT activity, only 5 have all four residues of the DEDD motif, which is responsible for coordinating the metals required for 3′–5′ exonuclease activity [65]. These bacterial species also maintain the same base-stacking residue, a tyrosine that helps orient the nucleotide at the 3′ end of the substrate [65]. The remaining species are predicted to have catalytically inactive 3′–5′ exonuclease domains and have a mix of tyrosine or histidine residues at the base-stacking position (Supplementary Table S1). Strikingly, the majority of Pol Is studied originate from extremophiles [40, 41]. As these proteins are stable at higher temperatures, the investigation of thermophilic Pol Is is most relevant for commercial applications necessitating specific *in vitro* conditions [40, 41]. Even prior work on *E. coli* Pol I have relied on Mn^2+^ to enhance activity [42]. Therefore, to understand the conservation of Pol I RT activity, we chose to explore versions of this enzyme from model organisms and bacterial pathogens, with a focus on proteins with catalytically inactive proofreading domains from several bacteria using *E. coli* Pol I for comparison. Now that we show *B. subtilis* Pol I to be an adept RT, we hypothesize that RT activity could be a conserved feature among Pol I enzymes.

To this end, we assayed a selection of Pol Is from *E. coli, S. aureus, Mycobacterium smegmatis*, and *Listeria monocytogenes*. All four proteins were expressed and purified from *E. coli* (Supplementary Fig. S1) and assayed using the same DNA–DNA and RNA–DNA templates as Fig. 2. The DNA polymerase activity of these proteins was confirmed using a DNA template, where all proteins show successful primer extension in either assay condition (Fig. 3A and B). When given an RNA template, the RT activities of the proteins varied. In the DNA polymerase buffer, *Bs* Pol I and *Sa* Pol I produced full-length product, while and *Ec* Pol I produced some full-length product (Fig. 3C). *Lm* Pol I and *Ms* Pol I produced small amounts of fully synthesized DNA in this buffer, with their major products stalling about halfway through synthesis (Fig. 3C). When the assays were performed using the RT-optimized buffer, all four polymerases were able to generate full-length products (Fig. 3D). When a longer RNA template was used, the results were similar (Fig. 3E and F). *Sa* Pol I and *Ec* Pol I behaved more similarly to *Bs* Pol I than *Lm* Pol I and *Ms* Pol I, but *Bs* Pol I consistently had fewer intermediate products remaining after 60 min of extension.

**Figure 3.**
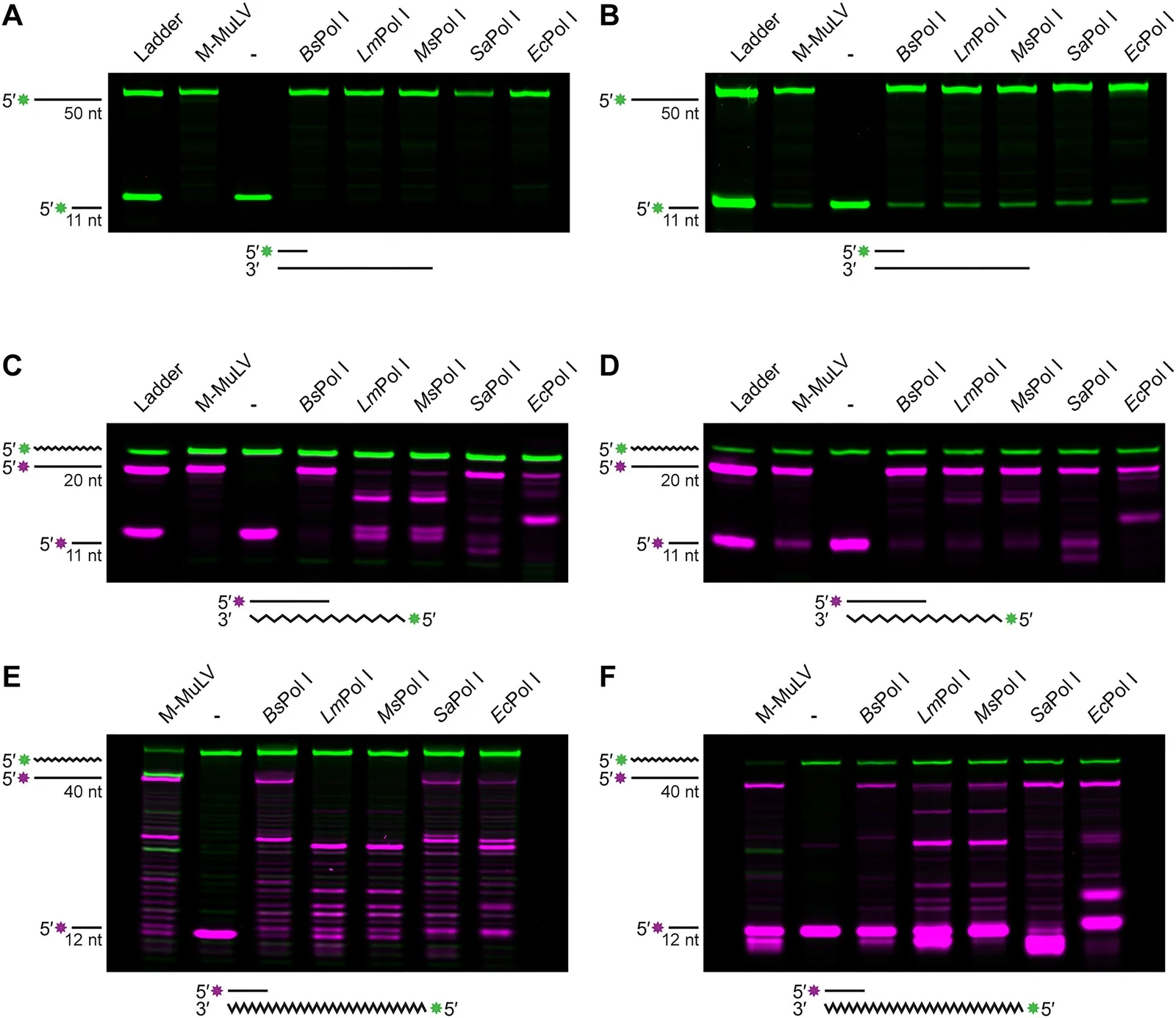
RNA-templated DNA repair is conserved in bacterial Pol I enzymes. (**A, B**) Primer extension using a 50-nt DNA template, (**C, D**), a 20-nt RNA template, and (**E, F**) a 40-nt RNA template. A, C, and E used a buffer optimized for DNA polymerase extension (Materials and methods). B, D, and F are the same, except reactions used a buffer optimized for RT activity (Materials and methods).

Previous work investigating *Ec* Pol I activity using an RNA template has led to inconsistent results, with some groups showing activity [42, 66–68] and other groups finding activity after protein modification or in the presence of high concentrations of Mn^2+^ [42, 46, 66]. Under our assay conditions, *Ec* Pol I was capable of RNA-templated DNA synthesis (Fig. 3). Since one of these studies used commercially available *Ec* Pol I-Klenow fragment and observed no RT activity [46], we compared the activity of commercially available *Ec* Pol I to the protein purified in this work and found that both produced similar results under our assay conditions (Supplementary Fig. S3). Therefore, we conclude that *Ec* Pol I is certainly capable of RNA-templated DNA synthesis, although the enzyme is not as efficient as *Bs* Pol I or *Sa* Pol I.

### Pol I and DnaE can traverse embedded ribonucleotides

Since all Pol I enzymes studied here are capable of catalyzing at least some RNA-mediated DNA synthesis, we chose to evaluate their ability to replicate a substrate that contained embedded ribonucleotides, as a model for a relevant circumstance encountered during DNA replication *in vivo*. We designed a series of substrates with a 15-nt primer-binding site followed by a 5-base “running start” of DNA. After the running start, Pol I would encounter 1, 5, 10, or 15 embedded rNMPs followed by another stretch of DNA (Fig. 4A). Both *Bs* Pol I and *Ec* Pol I could generate full-length products using all templates tested in both the extension (Ext.) and RT buffers (Fig. 4B and Supplementary Fig. S3), though the amount of intermediate-length product increased for each Pol I as the number of ribonucleotides increased, indicating both Pol Is lose efficiency as the ribonucleotide stretch increases. We chose to perform this assay for 20 min rather than 60 min because we found that *Ec* Pol I generates full-length substrate followed by cleavage using its 3′–5′ exonuclease activity within the extended timeframe (Supplementary Fig. S4). As Fig. 4C shows, the other Pol I homologs were not as robust across the different substrates as *Bs* and *Ec* Pol I, although they are clearly capable of traversing short patches of ribonucleotides. As before, the buffer composition impacted efficiency. The various Pol Is did produce more full-length product in the RT buffer than the DNA extension buffer (Fig. 4C and Supplementary Fig. S3).

**Figure 4.**
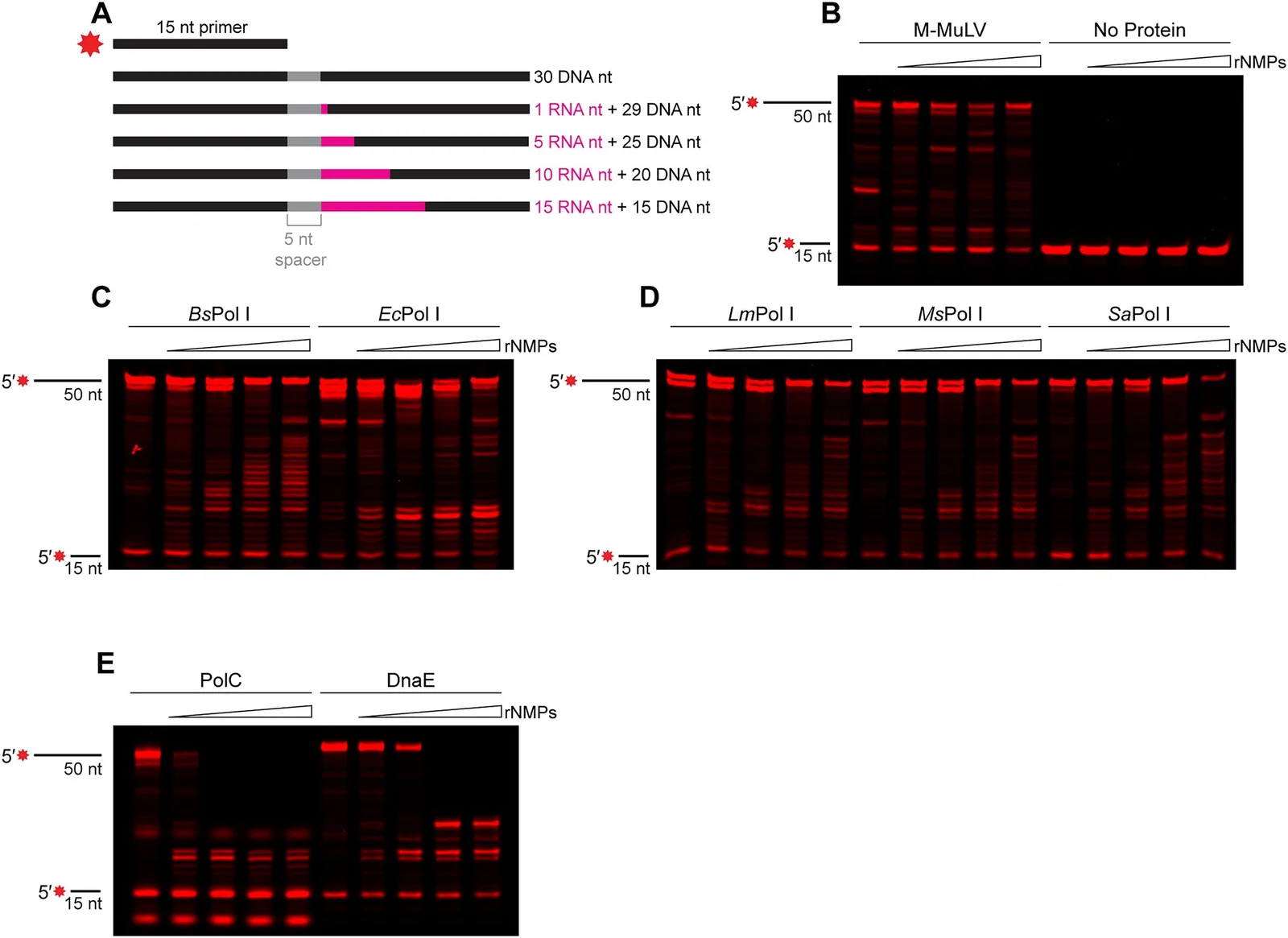
Pol I can traverse embedded ribonucleotides. (**A**) Schematic of substrates used to mimic ribopatches in DNA. (**B**–**E**) Primer extension products generated by the indicated polymerase after 20 min using substrates with increasing stretches of ribonucleotides. Assay performed with reverse transcription buffer (RT buffer).

Conversely, bacterial replicative C-family polymerases PolC and DnaE are expected to be strict DNA-dependent DNA polymerases [28, 29]. With that stated, to understand the potential division of labor for synthesis across rNMP errors, we asked if any number of template ribonucleotides could be tolerated by PolC and DnaE. PolC was able to fully extend the primer when the template was entirely composed of DNA or contained only one ribonucleotide (Supplementary Fig. S3). The ability to bypass this single rNMP required the specialized Ext. Buffer and was minimal in the more general RT buffer (Fig. 4E). PolC was unable to fully synthesize product when five or more ribonucleotide stretches were present in the template, demonstrating that embedded patches of rNMPs arrest replicative DNA polymerase PolC, which is the enzyme responsible for all leading and most lagging strand replication (Fig. 4E and Supplementary Fig. S3) [56]. Importantly, we find that DnaE was capable of traversing 1 or 5 rNMPs in the template DNA with a running start; however, longer stretches of ribonucleotides did arrest DnaE synthesis (Fig. 4E and Supplementary Fig. S3). Therefore, we find that PolC is most similar to replicative B-family eukaryotic DNA polymerases Pol δ and ε in ribonucleotide bypass ability [30–32], while DnaE is most analogous to eukaryotic polζ, a B-family polymerase, in ribonucleotide bypass activity [69]. We conclude that Pol I is efficient at RNA-templated DNA synthesis while DnaE is capable of tolerating short patches up to 5 ribonucleotides, suggesting that DnaE and Pol I provide two avenues to address 5 or fewer rNMPs in template DNA, with Pol I representing the most efficient and best option for longer stretches, establishing a DNA polymerase hierarchy for ribonucleotide bypass in *B. subtilis* (Fig. 4 and Supplementary Fig. S3). Further, our data show that the major leading and lagging strand replicative polymerase PolC experiences a near-complete arrest when more than one rNMP is encountered in an otherwise DNA template, demonstrating that this polymerase is poor at RNA-templated DNA synthesis and would require a switch to DnaE or Pol I for two or more rNMPs.

## Discussion

In this work, we demonstrate that DNA polymerase I and DnaE from *B. subtilis* exhibit DNA synthesis activity using RNA-containing templates. This activity allows DNA synthesis over both short and long stretches of embedded ribonucleotides to generate structures recognizable by RNase HII or HIII [62], providing cells with another chance to repair nested RNA before threatening genome stability. This is especially important following instances when intracellular dNTP pools are reduced, as such conditions increase the likelihood of rNMP incorporation by replicative or translesion DNA polymerases [25, 70]. Additionally, the significant RNA-templated synthesis of Pol I may enable this protein to specifically participate in RNA-mediated double-strand break repair [35, 46, 71]. We demonstrate that this RT activity is relevant to cells, showing that the loss of Pol I or Pol I DNA polymerase activity increases the HU sensitivity of cells lacking RNase HIII, a genotype that is prone to excessive R-loop formation and ribonucleotide incorporation [72], and providing a phenotype for Pol I RT activity. Together, these data show that Pol I provides a pathway for overcoming unresolved RNA–DNA hybrids. Further, these observations align with previous work suggesting that the major role of *Bs* Pol I is DNA repair [73–76] rather than Okazaki fragment maturation [54]. As such, we suggest that an important contribution of Pol I is its ability to traverse embedded ribonucleotides encountered by the replication fork, helping to ensure the integrity of the chromosome during replication.

In contrast to Pol I, neither PolC nor DnaE had substantial RT activity from a template composed entirely of RNA. When the rNMPs were embedded within DNA, PolC could traverse a single rNMP, but consecutive rNMPs arrested synthesis. Given the high incidence of single ribonucleotide misincorporations and the pausing of PolC at rNMPs [25], this tolerance demonstrates the necessary balance between processivity and accuracy. Importantly, we discovered that DnaE is capable of synthesizing DNA from a “running start” template containing a stretch of up to 5 rNMPs. This result demonstrates that DnaE is sufficient for synthesizing over short stretches of ribonucleotides during replication, but not for longer stretches of incorporated ribonucleotides due to integration from R-loops. Importantly, our result with DnaE provides evidence that at least one C-family DNA polymerase is able to synthesize short stretches of DNA from an RNA template, an activity that had not been observed prior. The limit of DnaE to stretches of 5 rNMPs further highlights the importance of Pol I’s significant RT activity for addressing longer patches of RNA embedded in template DNA.

Using standardized assay conditions, we also show that the RNA-templated synthesis activity of *Bs* Pol I is generally shared among bacterial Pol Is, though it is notable that *Bs* Pol I performs RT activity robustly under remarkably different assay conditions. The other Pol Is we investigate show enhanced activity in a buffer optimized for RT activity, an observation that offers some explanation as to the historical inconsistent characterization of the RT activity of *Ec* Pol I. Assay conditions vary across prior work, which mean some studies have reported minimal RT activity only under high Mn^2+^ conditions or after protein mutagenesis [42, 46], while others report activity in the absence of these factors [67, 68]. The consistently high activity of *Bs* Pol I indicates that this polymerase is particularly amenable to non-canonical templates, which is beneficial for a protein whose major role is in DNA repair.

The conservation of RT activity across diverse Pol Is, regardless of their proofreading capabilities, suggests that this function is more broadly modulated. While one group showed different extension from an RNA template using *Ec* PolA-Klenow and *Ec* PolA-Klenow^exo−^ [46], we find RNA-templated synthesis by the full-length version of *Ec* Pol I. The differences between our work and the prior study likely reflect differences in experimental conditions. The most obvious difference is higher ionic conditions in our experiments. Our results do show that the efficiency of the RNA-templated synthesis varies across organisms, and that *Bs* Pol I is an overlooked candidate for determining the structural basis of such disparity. As there are many commercial applications for a DNA polymerase capable of DNA/RNA mediated synthesis, most efforts to elucidate the elements affecting RT activity of these polymerases have focused on improving the activity. In pursuit of such modifications, it has been suggested that the Q-helix, which is a part of the polymerase “fingers” that interact with the template strand, or select residues of the “thumb” domain [77] may contribute to the adaptability of Pol Is active site. Since A-family polymerases are hypothesized to have evolved from RT [78–80], differences in active site flexibility may represent broader evolutionary adaptations from a common origin.

With our demonstration that DnaE, a C-family DNA polymerase, is able to traverse up to 5 embedded rNMPs, we can now conclude that at least one DNA polymerase from each of the five major polymerase families *(*A, B, C, X, and Y) has been shown to bypass a template with at least one ribonucleotide (Supplementary Table S5) [31, 32, 40, 47, 69, 81–83]. There is a substantial difference, however, between bypassing one ribonucleotide in an otherwise DNA template and usage of a primed RNA template for DNA synthesis, with the latter providing a more stringent test of RT activity. For example, we show that DnaE can bypass five rNMPs, yet DnaE is unable to synthesize from a primed RNA template. The DNA polymerases that have been shown to have more substantial RT activity using a complete RNA template include A-family polymerases Pol I, human Pol θ, and human Pol γ [42, 46, 47, 84, 85], human B-family polymerase Pol ζ, and Y-family polymerases mycobacterial DinB2 and archaeal Dpo4 [7, 86]. Other DNA polymerases tested across the five major families show much more limited usage of RNA templates (Supplementary Table S5). Notably, DNA polymerases from the A-family show the most consistent RT activity, which may be an evolutionary remanent, given that the A-family is proposed to have evolved from RT enzymes [78–80]. A caveat is that the amount of RT activity measured varies considerably within this group, as Pol I enzymes from thermophilic bacteria have been shown to possess wildly different synthesis activities [40]. This suggests that lineage alone is not sufficient to determine RT activity, as orthologous enzymes can demonstrate different levels of activity. Indeed, we present evidence that this inter-species variation in RT activity is an element that is present even among Pol I enzymes from non-thermophilic bacteria. Therefore, we suggest that the ability of DNA polymerases to bypass rNMPs in the template strand or their ability to complete stringent RT activity will vary from enzyme to enzyme and from species to species, perhaps depending on the intrinsic need for such an activity. In the case of *B. subtilis*, DnaE provides an avenue to bypass shorter rNMP patches, while Pol I enables synthesis over longer RNA patches. We suspect that each organism will have its own hierarchy for RNA bypass to tolerate short RNA patches of 1, 5 and longer patches >5. Given what is known (Supplementary Table S5), we predict that most replicative enzymes can bypass a single template rNMP, though cells may rely on B-, C-, or Y-family polymerases for up to 5 and perhaps use an A-, Y-, or B-family polymerase for >5 consecutive rNMPs. The specific DNA polymerases used in each scenario will depend on the organism tested.

In summary, our results demonstrate that Pol I is capable of RNA bypass synthesis, and we provide evidence that RT activity by Pol I contributes to genome stability under conditions that increase the occurrence of RNA–DNA hybrids. We show that, of the two C-family replicative DNA polymerases, PolC can bypass 1 rNMP and DnaE can bypass up to 5. Collectively, our study establishes the DNA polymerase usage for rNMP bypass in *B. subtilis*. These findings highlight the importance of diversity in dealing with a category of damage as broad, persistent, and ubiquitous as RNA–DNA hybrids.

## Supplementary Material

gkag971_Supplemental_File

## Data Availability

The data underlying this article are available in the article and in its online supplementary material.
