# Supplementary material for: DNA polymerase I and DnaE mediate RNA-templated DNA synthesis over embedded ribonucleotides
Source: Nucleic Acids Res. 2026 Oct 8;54(18):gkag971. doi: 10.1093/nar/gkag971 (PMC13646829; doi:10.1093/nar/gkag971)
Supplement: Supplementary file 1 [file gkag971_supplemental_file.pdf]

Supplementary Information

DNA polymerase I and DnaE mediate RNA-templated DNA synthesis over embedded ribonucleotides

Frances C. Lowder, Abigail H. Kendal, and Lyle A. Simmons*

Department of Molecular, Cellular, and Developmental Biology, University of Michigan, Ann Arbor, MI 48109

*LAS: Department of Molecular, Cellular, and Developmental Biology, University of Michigan, Ann Arbor, Michigan 48109-1055, United States. Phone: (734) 763-7142, Fax: (734) 647-0884 E-mail: lasimm@umich.edu

Short title: DNA polymerase hierarchy for replication of RNA templates

Keywords Reverse transcriptase, *Bacillus subtilis*, RNA-DNA hybrid, genome instability, DNA polymerase I

Supplemental Table 1. *B. subtilis* strains used in this study.

Strain Identifier	Relevant Genotype	Citation
FCL3	Δ*polA*	(1)
FCL10	native PY79	(2)
FCL11	Δ*rnhC*	(1)
FCL12	Δ*rnhC*, Δ*polA*	(1)
FCL263	*amyE* P_{spank}*::polA D834N*	This work
FCL264	Δ*polA, amyE* P_{spank}*::polA D834N*	This work
FCL265	Δ*rnhC, amyE* P_{spank}*::polA D834N*	This work
FCL266	Δ*polA*, Δ*rnhC, amyE* P_{spank}*::polA D834N*	This work

Supplemental Table 2. Oligonucleotides used in this study.

Oligonucleotide	Purpose	Sequence (5′-3′)
oJR46	Amplifying pE-SUMO vector (F)	TCGAGCACCACCACCACCACCACTGAG
oJR47	Amplifying pE-SUMO vector (R)	ACCTCCAATCTGTTCGCGGTGAGCCTCAATAATATCG
prFCL120	Amplifying *SapolA* (F)	ctcaccgcgaacagattggaggtGTGAATAAATTAGTATTAATCGATGG
prFCL121	Amplifying *SapolA* (R)	gtggtggtggtggtgctcgaTTATTTTGCATCATACCAGGTTGCACC
prFCL122	Amplifying *EcpolA* (F)	ctcaccgcgaacagattggaggtATGGTTCAGATCCCCCAAAATCCACTTATCC
prFCL123	Amplifying *EcpolA* (R)	gtggtggtggtggtgctcgaTTAGTGCGCCTGATCCCAGTTTTCG
prFCL124	Amplifying pTwist Insert (F)	GGCTCACCGCGAACAGATTGGAGGT
prFCL125	Amplifying pTwist Insert (R)	CAGTGGTGGTGGTGGTGGTGCTCGA
prFCL182	Mutating *polAD834N* (F)	GTACACaAcGAATTGATTTTTGAGGCG
prFCL183	Mutating *polAD834N* (R)	gTtGTGTACTTGAAGCAGAAGCCTTGC
oFCL7	Assay – DNA Template	GCA*A*T*CGACTCGTAAGCATGGTTCACTACTCGCTGCTTGATGCTCAATCG
oFCL8	Assay – DNA Primer	/5IRD800/C*G*A*TTGAGCATCAAGCAGCG
oFCL16	Assay – Short RNA Primer	/5IRD700/G*C*A*GAGCTAGC
oFCL18	Assay – Long RNA Primer	/5IRD700/A*C*A*GCGTTCCCT
oFCL20	Assay – Short RNA Ladder	/5IRD700/GCAGAGCTAGCTTACGATCG
oFCL21	Assay – Hybrid Primer	/5IRD700/C*A*A*GTCATCAAATGG
oFCL22	Assay – Hybrid DNA Template	TGAGTAAGTTGGTATCCGAGGTACTATGAGCTTCTGGACCATTTGATGACTTG
oFCL23	Assay – Hybrid 1 nt Template	TGAGTAAGTTGGTATCCGAGGTACTATGAGCTUCTGGACCATTTGATGACTTG
oFCL24	Assay – Hybrid 5 nt Template	TGAGTAAGTTGGTATCCGAGGTACTATGAGCUUCTGGACCATTTGATGACTTG
oFCL25	Assay – Hybrid 10 nt Template	TGAGTAAGTTGGTATCCGAGGTACUAUGAGCUUCTGGACCATTTGATGACTTG
oFCL26	Assay – Hybrid 15 nt Template	TGAGTAAGTTGGTATCCGAGGUACUAUGAGCUUCTGGACCATTTGATGACTTG
oFCL27	Assay – DNA Ladder	/5IRD800/C*G*A*TTGAGCATCAAGCAGCGAGTAGTGAACCATGCTTACGAGTCGATTGC
oJR227	Assay – Short RNA Template	/5IRD800CWN/CGAUCGUAAGCUAGCUCUGC
oJR336	Assay – Long RNA Template	/5IRD800CWN/CUGGAGGAUGGAGGAUGGUGGAGAUGUGAGGGAACGCUGU

* indicates phosphorothioate linkages
IRD indicates an infrared dye at the 5′ end or 3′ end as indicated, 700 or 800 indicates the wavelength of excitation, and CWN indicates a NHS ester conjugation.
Red bases indicate ribonucleotides.

Supplemental Table 3. Plasmids used in this study.

**Plasmid Identifier**	**Vector**	**Insert**
pJR22	pE-SUMO	*BspolA*(3)
pFCL56	pE-SUMO	*SapolA*
pFCL57	pTwist-Kan	*LmpolA**
pFCL58	pTwist-Kan	MspolA*
pFCL59	pE-SUMO	*EcpolA*
pFCL61	pE-SUMO	*LmpolA**
pFCL62	pE-SUMO	*MspolA**
pFCL93	pDR110	*polAD834N*

***** indicates genes codon optimized for expression in *E. coli*

Supplemental Table 4. Bacterial Pol Is with reverse transcriptase activity

Organism	Gram-stain	D	E	D	Y-H	D	Active 3′-5′ Exonuclease?	Notes
Bacillus caldolyticus EA.1 (4)	Positive	-	E	-	H	-	No	
Bacillus subtilis	Positive	-	E	-	H	-	No	First studied in this work.
Caldibacillus cellulovorans CompA.2 (4)	Positive	-	E	-	Y	-	No	
Caldicellulosiruptor saccharolyticus Rt69B.1 (4)	Positive	-	-	-	-	-	No	
Caldicellulosiruptor saccharolyticus Tok13B.1 (4)	Positive	-	-	-	-	-	No	
Caldicellulosiruptor saccharolyticus Tok7B.1 (4)	Positive	-	-	-	-	-	No	
Dictyoglomus thermophilum Rt46B.1 (4)	Negative	-	-	D	H	-	No	
Escherichia coli (5-10)	Negative	D	E	D	Y	D	Yes	
Geobacillus stearothermophilus (11,12)	Positive	-	E	-	H	-	Yes	
Listeria monocytogenes	Positive	-	E	-	H	-	Yes	First studied in this work.
Micrococcus luteus (6)	Positive	-	-	-	-	-	No	
Mycolicibacterium smegmatis (13)	-	-	-	-	-	-	No	Bound 1 Mn^{2+} using non-conserved residues (13)
Pseudomonas aeruginosa	Negative	D	E	D	Y	D	Yes	
Shigella sonnei (14)	Negative	D	E	D	Y	D	Yes	
Staphylococcus aureus (14)	Positive	-	E	-	Y	-	No	
Streptococcus agalactiae (15)	Positive	-	E	-	H	-	No	
Streptomyces coelicolor (16)	Positive	-	-	-	-	-	No	
Thermoactinomyces vulgaris	Positive	-	E	-	H	-	No	
Clostridium thermosulfurogenes (4)	Positive	-	-	-	Y	-	No	
Thermoclostridium stercorarium (4)	Positive	-	-	-	Y	-	No	
Thermotoga neapolitana (4)	Negative	D	E	D	Y	D	Yes	Modified to lose 3′-5′ (4)
Thermus aquaticus (4,17,18)	Negative	-	-	-	-	-	No	
Thermus filiformis (4)	Negative	-	-	-	-	-	No	
Thermus thermophilus (4,19,20)	Negative	-	-	-	-	-	No	Activity studied with Mn^{2+}(19,20)
Vibrio cholerae non-O1	Negative	D	E	D	Y	D	Yes	

Note that the identity of the *E. coli* hybrid polymerase purified by Lee-Huang and Cavalieri is unclear (9), but the reference is included in Sup. Table 1 since other works studying *Ec*Pol I make reference to it (7).

Supplemental Table 5. DNA polymerases tested on ribonucleotides for bypass activity.

DNA polymerase	DNA polymerase family	Type of organism	Ribonucleotide bypass activity	Notes and conditions	Citation(s)
DNA polymerase I	A	Bacteria	Highly variable <10 to >500	Dependent on the Pol I used and the experimental conditions.	(4,8,10,11,19)
DNA polymerase θ	A	Human	~11-17 rNMPs		(10,21,22)
DNA polymerase γ	A	Human	~20 rNMPs		(23,24)
DNA polymerase ν	A	Human	~7 rNMPs		(22)
DNA polymerase ζ	B	Yeast	~1-4 rNMPs<br>~30 rNMPs		(25,26)
DNA polymerase δ	B	Yeast	~1 rNMP		(25,27)
DNA polymerase δ	B	Human	~1 rNMP	10% bypass of 4 consecutive	(28)
DNA polymerase ε	B	Yeast	~1 rNMP		(29)
DnaE	C	*B. subtilis*	~5 NMPs		This work
Dpo4	Y	Archaea	41 rNMPs		(30)
DinB2	Y	Mycobacterium	~12 rNMPs		(31)
DNA polymerase η	Y	Human	1 rNMP		(32-34)
DNA polymerase κ	Y	Human	1 rNMP	Bypass by Pol κ is weak compared with Pol η	(35)
DNA polymerase ι	Y	Human	~1 rNMP	Less activity than pol κ or η	(22)
DNA polymerase β	X	Human	~1 rNMP	Gap filling	(22)
DNA polymerase λ	X	Human	~1 rNMP	Gap filling	(22)

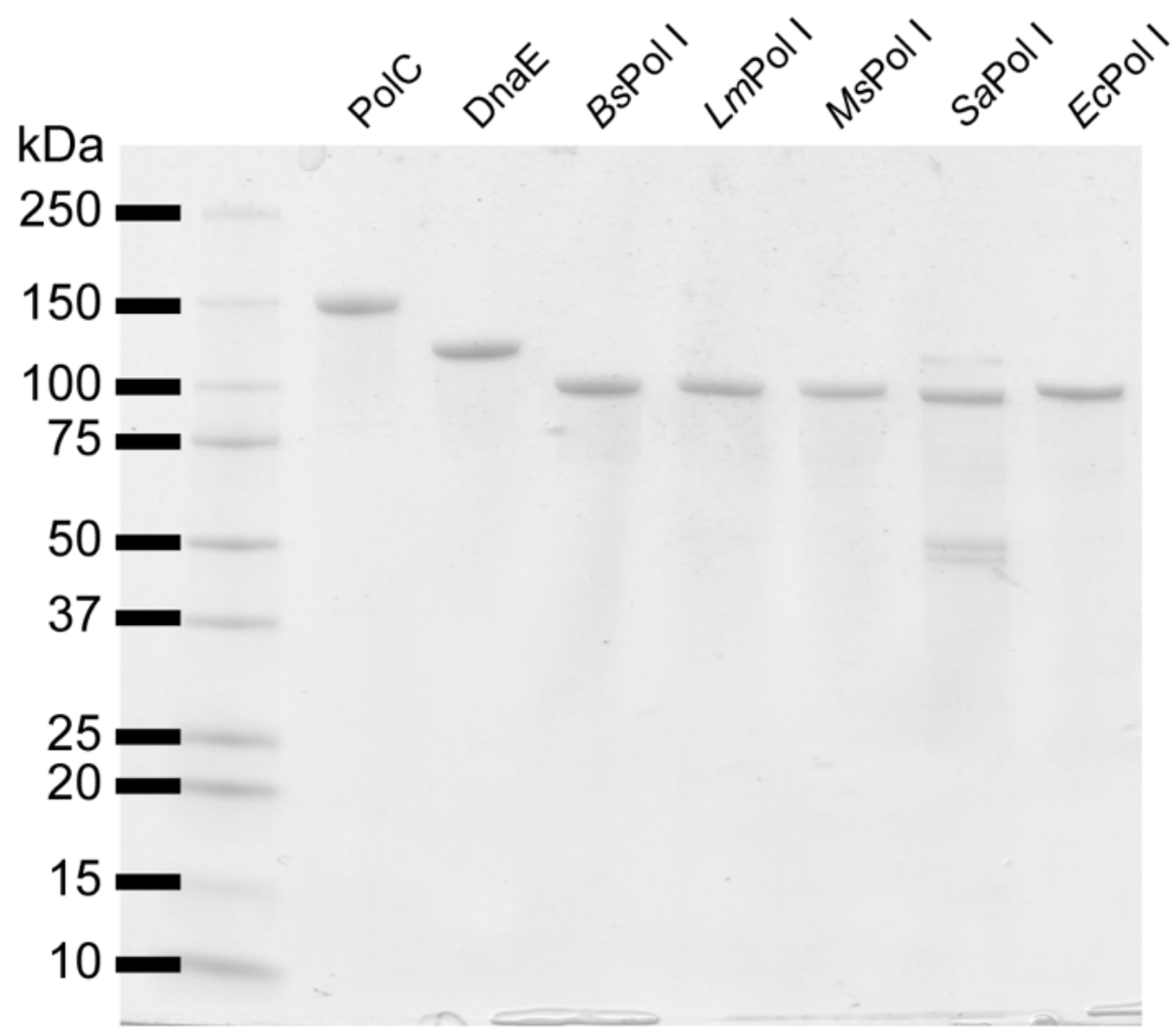

Sup. Figure 1. Purified proteins used in biochemical assays. 2 µg of each protein used for extension assays are shown on a Coomassie-stained, SDS-PAGE.

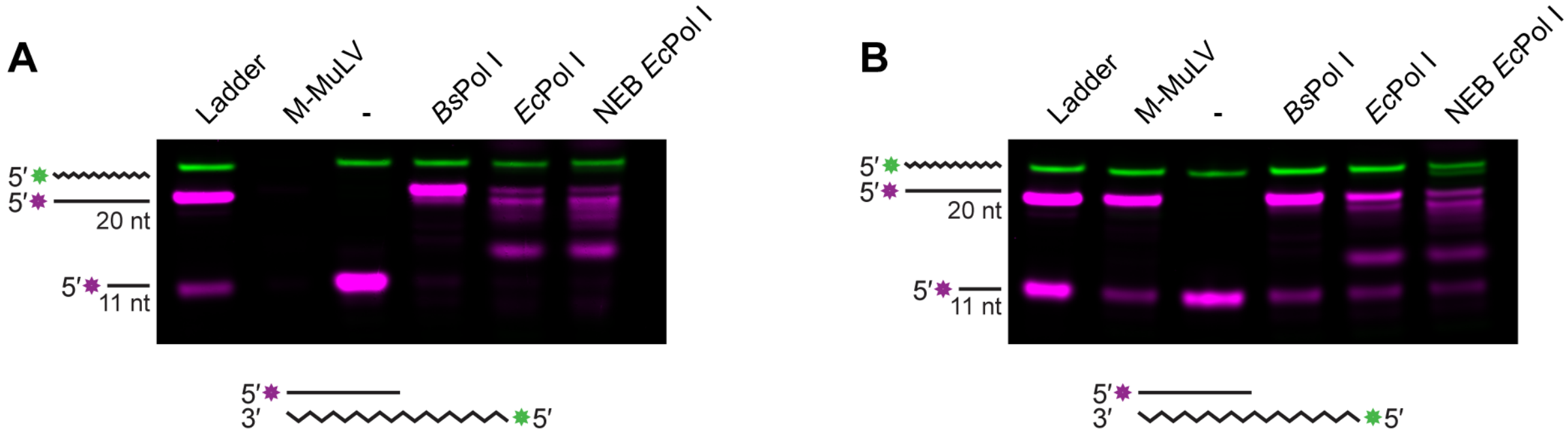

Sup. Figure 2. Commercial EcPol I has reverse transcriptase activity. (A) Primer extension in DNA extension buffer using an RNA template. (B) Primer extension from an RNA template in reverse transcriptase buffer.

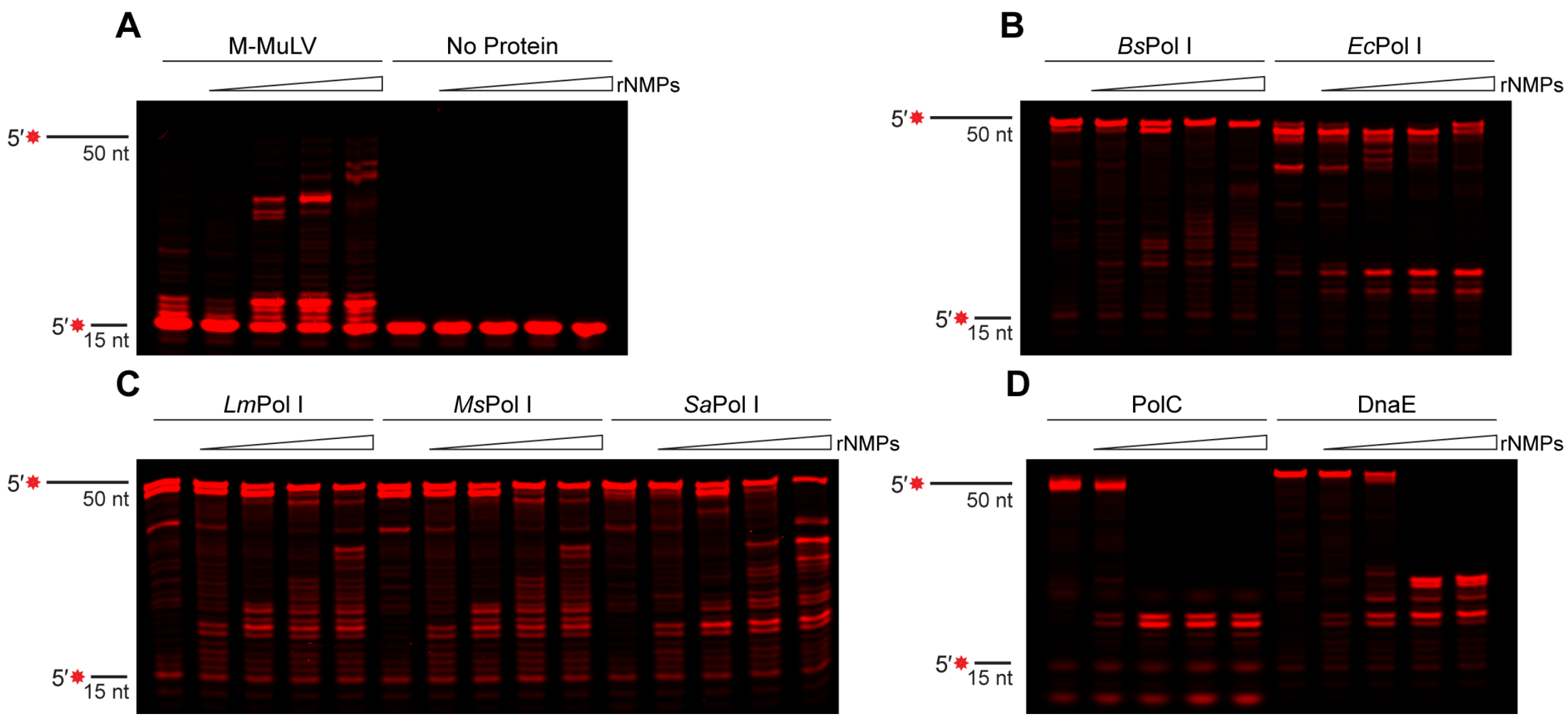

Sup. Figure 3. Pol Is traverse embedded ribonucleotides under DNA extension conditions. (A-D) Primer extension products generated by the indicated polymerase after 20 minutes using substrates with increasing stretches of ribonucleotides.

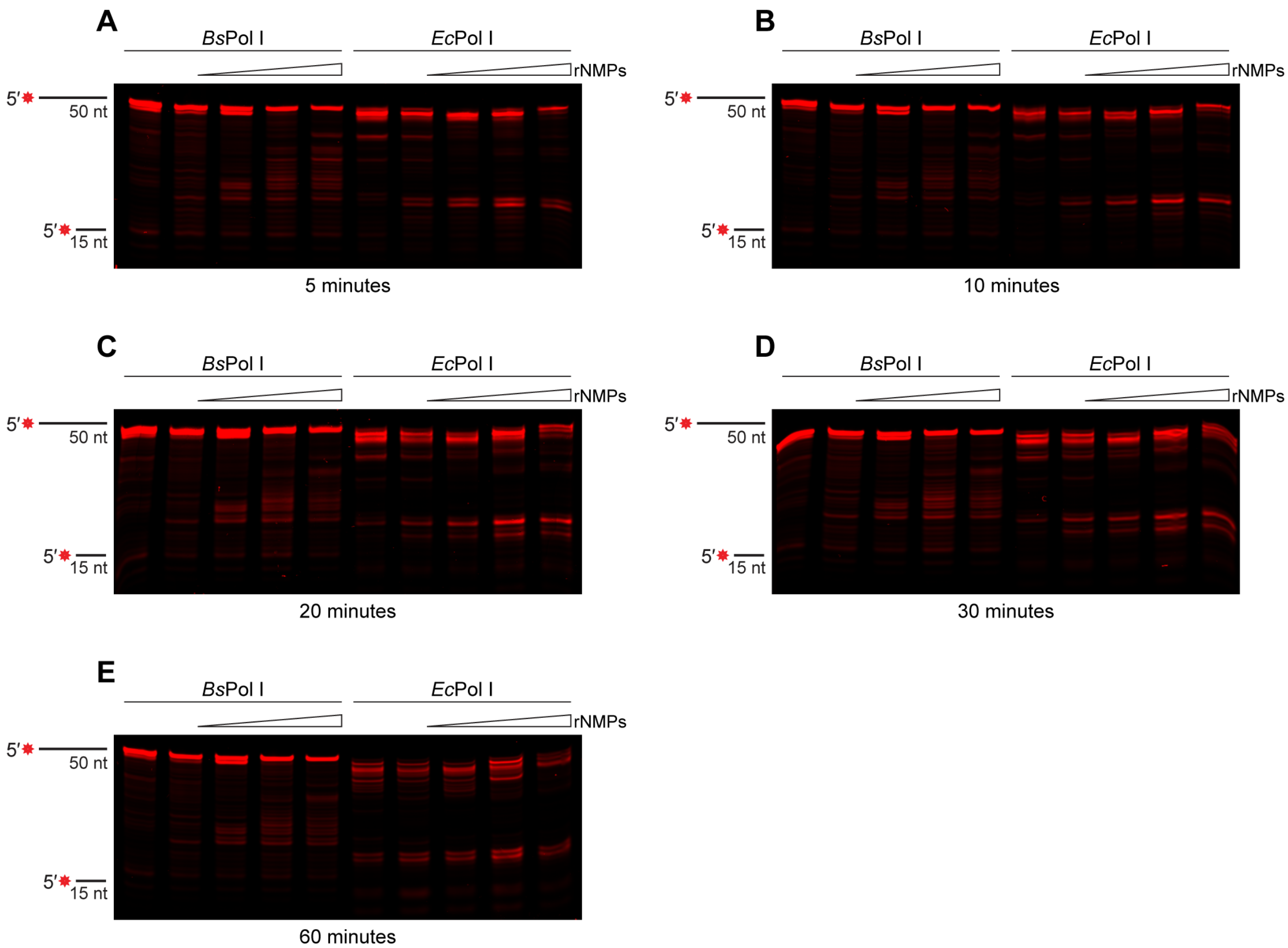

Sup. Figure 4. EcPol I nuclease activity degrades reaction products over time. (A-E) Primer extension products generated by the indicated polymerase using substrates with increasing stretches of ribonucleotides. Reaction incubation times are indicated below each gel.

References

1. Randall, J.R., Nye, T.M., Wozniak, K.J. and Simmons, L.A. (2019) RNase HIII Is Important for Okazaki Fragment Processing in *Bacillus subtilis*. *J Bacteriol*, **201**, pii: e00686–00618.
2. Youngman, P., Perkins, J.B. and Losick, R. (1984) Construction of a cloning site near one end of Tn917 into which foreign DNA may be inserted without affecting transposition in *Bacillus subtilis* or expression of the transposon-borne erm gene. *Plasmid*, **12**, 1–9.
3. Schroeder, J.W., Randall, J.R., Hirst, W.G., O'Donnell, M.E. and Simmons, L.A. (2017) Mutagenic cost of ribonucleotides in bacterial DNA. *Proc Natl Acad Sci U S A*, **114**, 11733–11738.
4. Shandilya, H., Griffiths, K., Flynn, E.K., Astatke, M., Shih, P.J., Lee, J.E., Gerard, G.F., Gibbs, M.D. and Bergquist, P.L. (2004) Thermophilic bacterial DNA polymerases with reverse-transcriptase activity. *Extremophiles*, **8**, 243–251.
5. Karkas, J.D., Stavrianopoulos, J.G. and Chargaff, E. (1972) Action of DNA polymerase I of *Escherichia coli* with DNA-RNA hybrids as templates. *Proc Natl Acad Sci U S A*, **69**, 398–402.
6. Wells, R.D., Flugel, R.M., Larson, J.E., Schendel, P.F. and Sweet, R.W. (1972) Comparison of some reactions catalyzed by deoxyribonucleic acid polymerase from avian myeloblastosis virus, *Escherichia coli*, and *Micrococcus luteus*. *Biochemistry*, **11**, 621–629.
7. Loeb, L.A., Tartof, K.D. and Travaglini, E.C. (1973) Copying natural RNAs with *E. coli* DNA polymerase I. *Nat New Biol*, **242**, 66–69.
8. Ricchetti, M. and Buc, H. (1993) *E. coli* DNA polymerase I as a reverse transcriptase. *EMBO J*, **12**, 387–396.
9. Lee-Huang, S. and Cavalieri, L.F. (1963) Polyribonucleotides as Templates for Polydeoxyribonucleotides. *Proc Natl Acad Sci U S A*, **50**, 1116–1122.
10. Chandramouly, G., Zhao, J., McDevitt, S., Rusanov, T., Hoang, T., Borisonnik, N., Treddinick, T., Lopezcolorado, F.W., Kent, T., Siddique, L.A. *et al.* (2021) Poltheta reverse transcribes RNA and promotes RNA-templated DNA repair. *Sci Adv*, **7**.
11. Shi, C., Shen, X., Niu, S. and Ma, C. (2015) Innate Reverse Transcriptase Activity of DNA Polymerase for Isothermal RNA Direct Detection. *J Am Chem Soc*, **137**, 13804–13806.
12. Jackson, L.N., Chim, N., Shi, C. and Chaput, J.C. (2019) Crystal structures of a natural DNA polymerase that functions as an XNA reverse transcriptase. *Nucleic Acids Res*, **47**, 6973–6983.
13. Ghosh, S., Goldgur, Y. and Shuman, S. (2020) Mycobacterial DNA polymerase I: activities and crystal structures of the POL domain as apoenzyme and in complex with a DNA primer-template and of the full-length FEN/EXO-POL enzyme. *Nucleic Acids Res*, **48**, 3165–3180.
14. Harada, F., Nakano, T., Kohno, T., Mohan, S. and Taniguchi, H. (2005) RNA-dependent DNA polymerase (RT) activity of bacterial DNA polymerases. *Bull Osaka Med Coll*, **51**, 35–41.
15. Bahrami, F. and Jestin, J.L. (2008) *Streptococcus agalactiae* DNA polymerase I is an efficient reverse transcriptase. *Biochimie*, **90**, 1796–1799.

16. Bao, K. and Cohen, S.N. (2004) Reverse transcriptase activity innate to DNA polymerase I and DNA topoisomerase I proteins of Streptomyces telomere complex. *Proc Natl Acad Sci U S A*, **101**, 14361–14366.
17. Jones, M.D. and Foulkes, N.S. (1989) Reverse transcription of mRNA by Thermus aquaticus DNA polymerase. *Nucleic Acids Res*, **17**, 8387–8388.
18. Barnes, W.M., Zhang, Z. and Kermekchiev, M.B. (2020) A Single Amino Acid Change to Taq DNA Polymerase Enables Faster PCR, Reverse Transcription and Strand-Displacement. *Front Bioeng Biotechnol*, **8**, 553474.
19. Myers, T.W. and Gelfand, D.H. (1991) Reverse transcription and DNA amplification by a Thermus thermophilus DNA polymerase. *Biochemistry*, **30**, 7661–7666.
20. Cai, D., Behrmann, O., Hufert, F., Dame, G. and Urban, G. (2018) Capacity of rTth polymerase to detect RNA in the presence of various inhibitors. *PLoS One*, **13**, e0190041.
21. Carvajal-Maldonado, D., Li, Y., Returan, M., Averill, A.M., Doublie, S. and Wood, R.D. (2024) Dynamic stem-loop extension by Pol theta and templated insertion during DNA repair. *J Biol Chem*, **300**, 107461.
22. Tsegay, P.S., Hernandez, D., Qu, F., Olatunji, M., Mamun, Y., Chapagain, P. and Liu, Y. (2023) RNA-guided DNA base damage repair via DNA polymerase-mediated nick translation. *Nucleic Acids Res*, **51**, 166–181.
23. Lee, H.R. and Johnson, K.A. (2007) Fidelity and processivity of reverse transcription by the human mitochondrial DNA polymerase. *J Biol Chem*, **282**, 31982–31989.
24. Murakami, E., Feng, J.Y., Lee, H., Hanes, J., Johnson, K.A. and Anderson, K.S. (2003) Characterization of novel reverse transcriptase and other RNA-associated catalytic activities by human DNA polymerase gamma: importance in mitochondrial DNA replication. *J Biol Chem*, **278**, 36403–36409.
25. Lazzaro, F., Novarina, D., Amara, F., Watt, D.L., Stone, J.E., Costanzo, V., Burgers, P.M., Kunkel, T.A., Plevani, P. and Muzi-Falconi, M. (2012) RNase H and postreplication repair protect cells from ribonucleotides incorporated in DNA. *Molecular Cell*, **45**, 99–110.
26. Mayle, R., Holloman, W.K. and O'Donnell, M.E. (2024) DNA polymerase zeta has robust reverse transcriptase activity relative to other cellular DNA polymerases. *J Biol Chem*, **300**, 107918.
27. Yao, N.Y., Schroeder, J.W., Yurieva, O., Simmons, L.A. and O'Donnell, M.E. (2013) Cost of rNTP/dNTP pool imbalance at the replication fork. *Proceedings of the National Academy of Sciences of the United States of America*, **110**, 12942–12947.
28. Clausen, A.R., Zhang, S., Burgers, P.M., Lee, M.Y. and Kunkel, T.A. (2013) Ribonucleotide incorporation, proofreading and bypass by human DNA polymerase delta. *DNA Repair (Amst)*, **12**, 121–127.
29. Watt, D.L., Johansson, E., Burgers, P.M. and Kunkel, T.A. (2011) Replication of ribonucleotide-containing DNA templates by yeast replicative polymerases. *DNA Repair (Amst)*, **10**, 897–902.

30. Wang, M., Jiang, W., Liu, X., Wang, J., Zhang, B., Fan, C., Liu, L., Pena-Alcantara, G., Ling, J., Chen, J. *et al.* (2019) Mirror-Image Gene Transcription and Reverse Transcription. *Chem*, **5**, 848–857.
31. Ordonez, H., Uson, M.L. and Shuman, S. (2014) Characterization of three mycobacterial DinB (DNA polymerase IV) paralogs highlights DinB2 as naturally adept at ribonucleotide incorporation. *Nucleic Acids Res*, **42**, 11056–11070.
32. Su, Y., Egli, M. and Guengerich, F.P. (2017) Human DNA polymerase eta accommodates RNA for strand extension. *J Biol Chem*, **292**, 18044–18051.
33. Su, Y., Ghodke, P.P., Egli, M., Li, L., Wang, Y. and Guengerich, F.P. (2019) Human DNA polymerase eta has reverse transcriptase activity in cellular environments. *J Biol Chem*, **294**, 6073–6081.
34. Franklin, A., Milburn, P.J., Blanden, R.V. and Steele, E.J. (2004) Human DNA polymerase-eta, an A-T mutator in somatic hypermutation of rearranged immunoglobulin genes, is a reverse transcriptase. *Immunol Cell Biol*, **82**, 219–225.
35. Sassa, A., Caglayan, M., Rodriguez, Y., Beard, W.A., Wilson, S.H., Nohmi, T., Honma, M. and Yasui, M. (2016) Impact of Ribonucleotide Backbone on Translesion Synthesis and Repair of 7,8-Dihydro-8-oxoguanine. *J Biol Chem*, **291**, 24314–24323.
